# “Online, the counselor can't see me cry”: a systematic literature review on emotion and computer-mediated care

**DOI:** 10.3389/fdgth.2023.1216268

**Published:** 2023-09-01

**Authors:** Sarah De Coninck, Elke Emmers

**Affiliations:** ^1^ Research Unit Inclusive Society, University College Leuven Limburg, Leuven, Belgium; ^2^ Research Units Sustainable Resources and Smart Organizations, University College Leuven Limburg, Diepenbeek, Belgium; ^3^ Brain and Cognition, KU Leuven, Leuven, Belgium; ^4^ School of Educational Studies, UHasselt, Hasselt, Belgium

**Keywords:** eHealth, computer-mediated care, emotion regulation, empathy, emotional support, emotion expression

## Abstract

**Introduction:**

Computer-mediated care is becoming increasingly popular, but little research has been done on it and its effects on emotion-related outcomes. This systematic literature review aims to create an overview that addresses the research question: “Is there a relationship between computer-mediated care and emotional expression, perception, and emotional and (long-term) emotion outcomes?”

**Method:**

This systematic literature review was conducted in accordance with the Preferred Reporting Items for Systematic Reviews and Meta-Analyses (PRISMA) guidelines and used five eligibility criteria, namely, (1) participants: adults seeking support; (2) intervention: eHealth; (3) diagnostic criteria: transdiagnostic concept of difficulty identifying, expressing, and/or regulating emotions (e.g., alexithymia); (4) comparator: either face-to-face care or no comparator; and (5) study design: quantitative studies or qualitative studies. Quality was assessed using the QualSyst tool.

**Results:**

The analysis includes 25 research papers. Self-paced interventions appear to have a positive effect on emotion regulation. Videoconferencing interventions improved emotion regulation from before to after treatment but worsened emotion regulation compared with face-to-face treatment.

**Discussion:**

The lack of variation in the modalities studied and the emotion measurements used make it difficult to draw responsible conclusions. Future research should examine how different modalities affect the real-time communication of emotions and how non-verbal cues influence this.

## 1. Introduction

The mediation of social and health services by computers and other digital devices is becoming increasingly popular (e.g., (1)). In 2017, over 10,000 mental health apps were available (2). Moreover, considering the COVID-19 pandemic, legislation has been rapidly changing to increase the availability of computer-mediated care (3, 4).

Available effectiveness research focuses mainly on care *via* videoconferencing and shows that it is feasible and effective for a wide range of psychopathologies (5–8). Two studies found a better treatment response or faster decrease in symptoms when treatment for children and their parents was delivered *via* the Internet compared with face-to-face (9, 10). The treatment response and the bond between a therapist and their client appear to be unchanged when video conferencing is compared with face-to-face interactions (11). Despite these findings, clinicians report a decrease in the ability to express empathy online (12). Both empathy skills and the number of empathic interactions appear lower in online sessions compared with face-to-face interactions (13, 14).

In their seminal work, Grondin et al. (15) developed a theoretical framework to elucidate the concept of computer-mediated empathy. This article follows the work of Grondin and colleagues and will discuss the phenomenon of computer-mediated empathy, which involves individuals seeking and/or providing support online. It will examine the dynamics between a counselor and their client, as well as the roles of the “target” and “observer” in this context. The concept of computer-mediated empathy encompasses a sequential interaction process comprising four distinct stages, namely, the transmission of social–emotional signals, the experience of empathy, the transfer of empathy, and the perception of empathy. The initiation of an empathic interaction involves the target expressing their emotional state to the observer. The extent to which non-verbal signals are filtered depends on the specific modality employed, such as videoconferencing or text-based communication. Therefore, it is necessary to modify the message to communicate emotions effectively. According to Barak et al. (16), the individual being studied has the option to explicitly express their emotional state by stating phrases such as “I'm crying” or “I feel sad.” In addition, textual paralanguage cues, such as symbols, images, or emoticons, can be employed to convey emotions (17–19). Furthermore, emotions can also be expressed using character repetitions or punctuation, as exemplified by phrases such as “This is taking soooooo loooooong,” [blank message], and “He never talks about IT” (18, 19).

Subsequently, the individual at the receiving end of the computer interface must adeptly discern the emotional state of the target and cultivate a sense of empathy. The subsequent phase entails the transmission of empathetic sentiments by the observer, as also outlined by Grondin et al. (15). The ability to convey empathy is influenced by both verbal and non-verbal cues, as suggested by previous research (20, 21). Therefore, filtering non-verbal cues affects one's ability to express empathy. Observers can directly inquire about the emotional state of the individual in question, specifically regarding feelings of sadness. However, they encounter limitations in effectively communicating subtle variations in facial expressions or body language. According to Grondin et al. (15), the target must interpret the response as empathetic. It is crucial to establish this differentiation when expressing empathy, as research has shown that the perceived level of empathy is a more accurate indicator of therapeutic outcomes compared with the observer's assessment of the therapist’s own experience of empathy (22).

**Figure 1:**
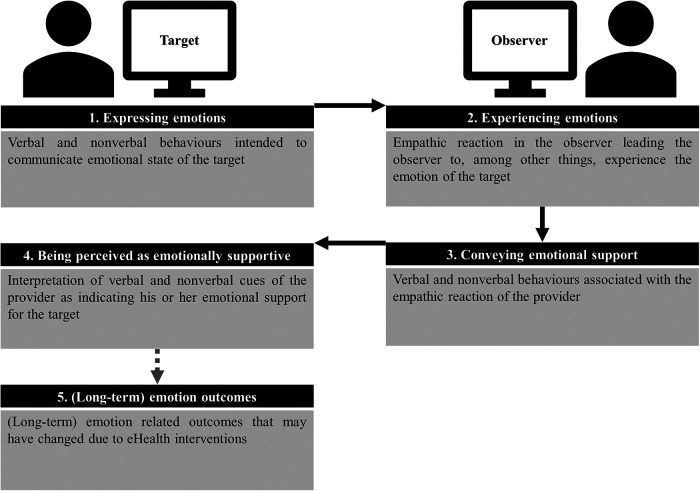
Framework for expressing and experiencing emotions and emotional support via computer-mediated interaction based on the framework of computer-mediated empathy by Grondin et al. (15).

Figure 1 shows the complicated target–observer relationship in computer-mediated care. The target, who needs emotional assistance, sends social–emotional signals using a computer. Their SMS messages may express their ideas, feelings, and experiences. “I feel overwhelmed with work deadlines” or “I'm going through a difficult time and need someone to talk to” are sample statements of emotional support requests. The observer, who provides empathy and emotional support, deciphers these social–emotional indicators and responds with compassion. Virtual hugs, encouraging words, and supporting texts convey this response. The observer may say, “I'm here for you. You can get through this,” or “I understand how difficult that must be for you.” The target's emotions are affected by the observer's empathy. Computer-mediated empathy can boost the target's mood with the observer's pleasant words. Computer-mediated empathy helps moderate interpersonal emotions even after participation. Zaki and Williams (23) found that computer-mediated routes can provide emotional support to the target.

Little is known about the ability to express and experience emotions and emotional support through computer-mediated care. An overview of the effects of computer-mediated communication on emotion-related outcomes is also lacking. Little is known about how other modalities influence emotion-related outcomes, given that most available research focuses on videoconferencing. Thus, this systematic literature review addresses the following research questions:

(1) Is there a link between computer-mediated care and (a) emotional expression, (b) emotional experience, and (c) emotional support during the interaction?
(2) Is there a link between computer-mediated care and emotion-related outcomes after the interaction?

## 2. Methods

The present study employed a systematic literature review approach, following a protocol developed *a priori*, adhering to the guidelines outlined in the Preferred Reporting Items for Systematic Reviews and Meta-Analyses (PRISMA). Five specific eligibility criteria were employed in the selection process. The assessment of quality was conducted utilizing the QualSyst tool.

### 2.1. Literature search

Papers were searched following a pre-developed protocol in EBSCO using keywords such as “eHealth” and “Emotio* identification” (for the complete search string, see Supplementary File S1, Table 1). All relevant databases (e.g., The Cochrane Library, Google Scholar, PubMed, PsychInfo, CINAHL, ERIC, ProQuest Dissertations, and EBSCO) were searched to include academic and gray literature. The reference lists of retrieved studies, (systematic) reviews, meta-analyses, and eHealth whitepapers and reports were searched as well. No ethnic or geographic limits were adopted. No client or ethical approval was required.

### 2.2. Screening and eligibility criteria

All studies have been screened for publication year (2000 to date) and adopted language (English or Dutch), and the records were deduplicated. The eligibility criteria include (1) participants: adults seeking support; (2) intervention: eHealth; (3) diagnostic criteria: transdiagnostic concept of difficulty identifying, expressing, and/or regulating emotions (e.g., alexithymia); (4) comparator: either face-to-face care or no comparator; and (5) study design: quantitative or qualitative studies. Quality was assessed using the QualSyst tool.

### 2.3. Data extraction

Data were extracted based on a pre-developed protocol. Study characteristics included the country in which the study was conducted, the intervention(s), the comparator(s), the target population, and the sample size. For each study, the instruments used for data collection, the method of delivery of the intervention, and the results of the measurements were reviewed to analyze the effect of the intervention.

### 2.4. Quality assessment

The two authors individually critically appraised the quality of the studies using the standard quality assessment criteria for evaluating primary research documents (QualSyst tool) (24). Supplementary File S4, Tables 4, 5 report two separate tables indicating the quality for the quantitative and qualitative studies.

## 3. Results

### 3.1. Literature search

**Figure 2:**
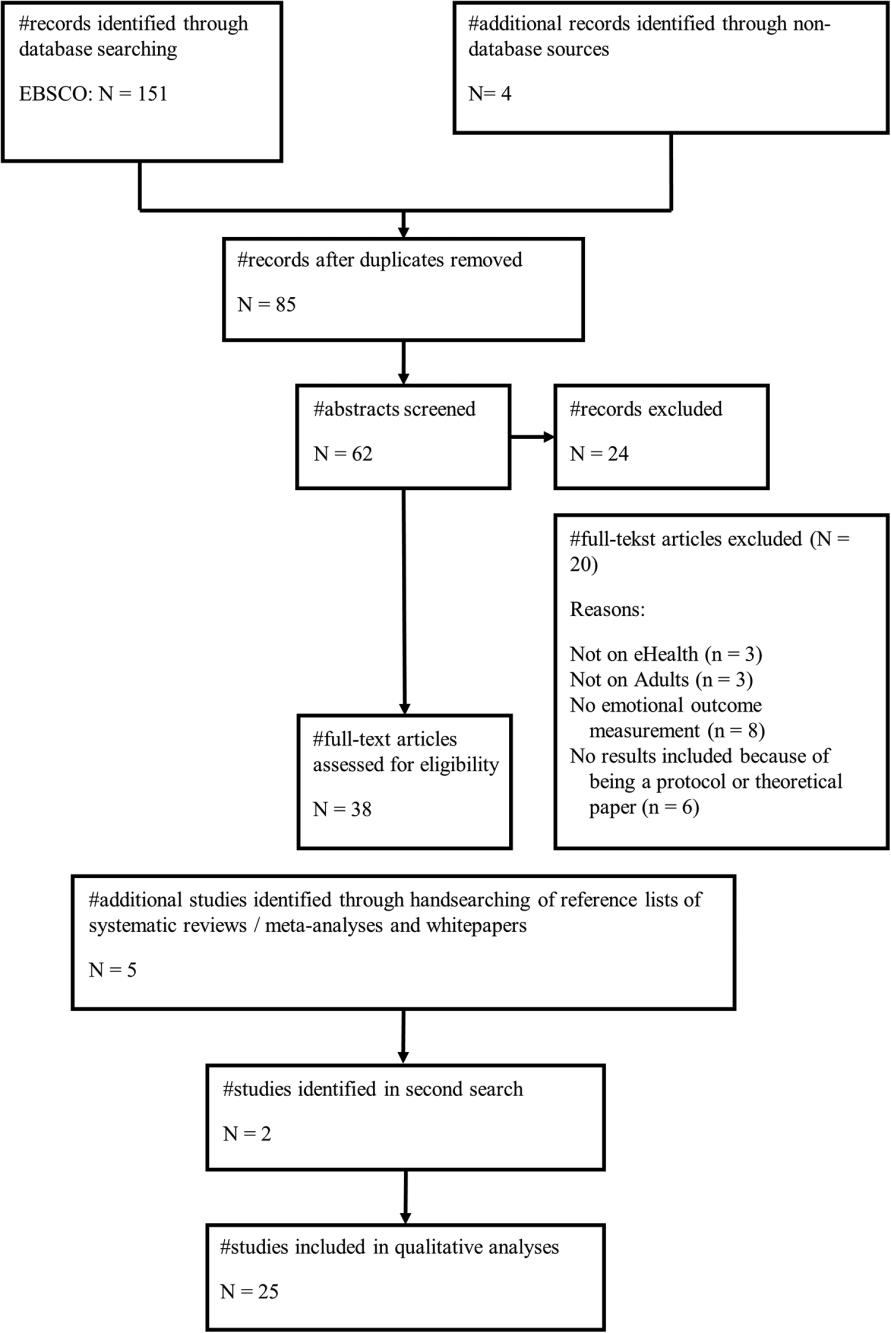
Flowchart of the review.

A total of 151 studies were identified based on the search string, with 66 duplicates excluded. The two reviewers screened 85 titles and abstracts independently. The selection underwent thorough approval by the author team, wherein any disagreements were addressed through constructive discussions, accompanied by well-founded arguments, ultimately leading to a consensus. The inter-assessor reliability was 0.70. Figure 2 shows the full flowchart of this systematic literature review. A total of 25 papers were included in the analysis.

### 3.2. Study characteristics

Included studies are internationally dispersed, with most of the studies originating in the USA (e.g., (25–27)) (see Supplementary File S2, Table 2). Some studies were conducted internationally through an online community (e.g., (28–30)).

Study designs include six qualitative studies (e.g., (25, 31)), three surveys (e.g., (32–34)), and one case study (35), but the majority of the studies are RCTs (e.g., (36–38)).

The study populations vary but have a predominant medical focus, such as breast cancer patients (e.g., (25, 26, 39)), traumatic brain injury patients (e.g., (40)), patients with mental health problems (e.g., (28, 41)), and employees (e.g., (42–44)).

### 3.3. Quality of the included studies

All 19 quantitative studies were of good quality overall. Results in Supplementary File S4, Table 4 show that the range is from 0.64 (min) to 1.00 (max), with an average score of 0.85, and show seven studies of good quality (0.70–0.90) (e.g., (26, 36, 37)) and nine studies with excellent quality (>0.90) (e.g., (27, 28, 42)).

The scores for the qualitative studies were calculated similarly (24). The quality of the qualitative studies is low, showing a minimum of 0.35 (e.g., (45, 46)) and a maximum of 0.80 (e.g., (31)), with a score average of 0.55 (see Supplementary File S4, Table 5).

### 3.4. Modalities used

There was significant variability in the modalities used in the included studies. Five studies used a synchronous communication mode (e.g., (26, 35, 37)), meaning that the interaction took place in real time, while the majority of studies used an asynchronous communication mode (e.g., (25, 31, 36)).

In total, 12 interventions were text-based (e.g., (25, 31, 47)), 5 made use of videoconferencing (e.g., (26, 35, 37)), and 8 used a communication mix (e.g., (28, 36, 38)).

### 3.5. Is there a connection between eHealth and emotional expression, experience, support, and (long-term) emotion outcomes?

#### 3.5.1. Expressing emotions

The studies on emotional expression mostly investigate text-based online support communities (e.g., (25, 31, 47)) (see Supplementary File S3, Table 3a). Qualitative analysis of messages posted in online support groups indicates that emotions are regularly expressed verbally in these communities (e.g., (25, 31, 47)). Online bereavement and cancer communities predominantly feature emotional communications (25, 45). While emotions are expressed online, it seems necessary to modulate the intensity of these emotions instead of presenting them in its raw form, for example, by using humor and storytelling (46) or sharing more positive than negative emotions (25, 27).

There seems to be a discrepancy between online and real-life emotional expressions. A questionnaire administered to participants in online breast cancer communities revealed that individuals expressed more emotions online than in face-to-face interactions (33). This finding is supported by a qualitative analysis of messages in an online support group for men with eating problems (47). This is in accordance with the online disinhibition effect, which claims that people are inclined to express themselves more openly online (48).

#### 3.5.2. Experiencing the emotions of the user on the other side of the screen

No studies have investigated whether users experienced the emotions of another user during computer-mediated interactions. However, one study indicates that using positive emotions is associated with a lower chance of response (27). Another study suggests that too much unmodulated emotion reduces group interaction and individual involvement (46) (see Supplementary File S3, Table 3b). This reduced interaction may indirectly indicate a difficulty in experiencing the emotions of the target after reading positive emotions or too much unmodulated emotion.

#### 3.5.3. Conveying emotional support

Two studies describe how emotional support can be provided, namely, by sharing certain feelings, supporting each other’s well-being, and showing sympathy, compassion, and understanding (31, 47). Moreover, empathy was the main emotion expressed in a bereavement community (45.6% (45)) (see Supplementary File S3, Table 3c).

#### 3.5.4. Perceived emotional support

Self-report surveys on online cancer communities showed that perceived emotional support led to less perceived life stress (34) and that participants experienced less emotional support online than in face-to-face interactions (33) (see Supplementary File S3, Table 3d).

#### 3.5.5. Emotion outcomes after the interaction

Results concerning emotion outcomes after the interaction are inconsistent and relate to different modalities of computer-mediated care and different emotion outcomes (see Supplementary File S3, Table 3e). One study found that the emotion perception of participants increased after web-based training when compared with a waitlist control group (49).

As mentioned earlier, men with eating disorders reported expressing fewer emotions face-to-face and instead sought anonymous support online (47). After spending more time in the online group, they reported becoming less secretive in real-life meetings, suggesting a possible effect of the online environment on emotional expression. In contrast, another study of a video conference group found no effect on emotional expression before and after participation (26).

Emotion regulation improved from pre- to post-treatment in individuals suffering from dissociative disorder (28) and women with a BMI over 25 (38) after a self-paced intervention. In addition, compared with a waitlist control, emotion regulation increased in stress-sensitive employees (42), women with postpartum depression (50), business students (49), and women with a BMI over 25 (38) following the same intervention. Moreover, a self-paced intervention for breast cancer patients decreased emotional suppression from pre- to post-treatment but had no effect on cognitive reappraisal (30). Employees with high-risk drinking experienced less emotional irritation compared with waitlist control after a web-based intervention (36). In contrast, employees with work-related stress showed less emotional exhaustion after a web-based intervention from pre- to post-treatment compared with waitlist control (44).

Contrary to these results, a self-paced web-based stress management intervention for employees showed no effects on emotion regulation (43), and a text-based cancer support group showed higher emotional suppression after the intervention (39).

Videoconferencing interventions resulted in a lower degree of reappraisal and higher emotional suppression compared with face-to-face treatment for persons with medically unexplained pain (37). However, for female veterans who have experienced military sexual trauma (41) and people with traumatic brain injuries (40), videoconferencing interventions improved post-treatment emotion-regulatory skills. In a case study involving a man suffering from intermittent explosive disorder, mindfulness-based cognitive behavioral therapy delivered *via* videoconferencing showed a decline in aggressive episodes (35).

## 4. Discussion

This systematic literature review aimed to investigate whether emotions can be expressed, experienced, and supported effectively through computer-mediated care and the effects thereof on emotion-related outcomes.

### 4.1. Context and quality of the studies

The study characteristics show significant variations in the international context, study design, and study population, making it impossible to draw overall conclusions. The majority of the studies originate in the USA (e.g., (25–27)), making it difficult to generalize results to other regions of the world.

Overall, all quantitative studies were of good quality, but the quality of the qualitative studies was low. In contrast to this finding, the ratings of quantitative and qualitative studies by the developers of Qualsyst seem to be relatively similar (24), suggesting that the qualitative studies included in this review are of poor quality (e.g., (45, 46)). Qualitative studies are gaining popularity because they provide an in-depth picture of social health problems and individual experiences. However, the lack of information about the study standards for qualitative studies in the health sector leads to poorly conducted, analyzed, or reported studies (51).

### 4.2. Emotion expression

The studies on emotion expression are all considered text-based online support communities (e.g., (31)) and describe how much of the conversation contains explicit emotional expression (e.g., (25)). Interestingly, there seems to be some support for a possible disinhibition effect (48), where anonymity lowers the threshold to share emotional experiences online (47). However, no studies investigate whether an observer on the other end of the computer recognizes emotion expression.

### 4.3. Experiencing emotional support

The studies on experiencing emotional support solely look at text-based message boards. While emotions are regularly expressed online, participants experience less emotional support compared with face-to-face interactions (33). This finding might indicate that computer-mediated empathy online is lacking beyond the initial expression of emotions.

### 4.4. Emotion outcomes after the interaction

The results concerning emotion outcomes after the interaction are quite heterogeneous. Different intervention modalities lead to different results on emotion expression, making it impossible to draw any general conclusions.

The results are somewhat more evident when looking at emotion regulation. Emotion regulation seems enhanced after going through self-paced online interventions (28, 30, 42, 50) and interventions *via* videoconferencing (e.g., (40)). However, comparisons with face-to-face treatment are still lacking. Only one study compared face-to-face treatment with videoconferencing, which found lower emotion regulation skills after the videoconferencing condition (37).

### 4.5. Modalities

It is difficult to compare the modalities of the included studies with those of other studies, as the field is rapidly evolving and different taxonomies exist (e.g., (6, 52)). However, keeping in mind the framework on computer-mediated empathy (15), the characteristics of the online modality that are most relevant for the expression and experience of emotions are (1) communication richness, (2) synchronicity of the modality, (3) transmission quality, and (4) communication content. While communication richness and synchronicity are specific to the modality used, transmission quality is specific to the internet connection, and communication content can be altered by user customization (e.g., using emoticons and avatars and adjusting the webcam position). Therefore, a description of the modality is insufficient to establish these characteristics for the described interventions. Moreover, some studies used a communication mix (e.g., (28, 36, 42, 50)), making it even more difficult to determine the influence of the modality used on emotional expression, experience, and support.

In addition, no studies on text-based interventions looked at the effects of using symbols, images, emoticons (17–19), or textual paralanguage cues (18, 19) to convey emotions, limiting the conclusions that can be drawn.

### 4.6. Limitations and future directions

The research discussed in this paper can yield significant findings regarding the correlation between computer-mediated care and emotional consequences. Nevertheless, it is imperative to acknowledge the presence of limitations. Firstly, this systematic literature review focused on the eligibility criteria, including adults seeking help with transdiagnostic emotional identification, expression, and regulation. The narrow focus of this study may limit its relevance to other demographics or emotional concerns. In addition, the study explored a restricted number of modalities, such as self-paced interventions and videoconferencing, so it may limit the variety of computer-mediated care options. Chat-based therapies and virtual reality therapy may create different emotional effects.

Furthermore, the available studies show methodological shortcomings. Few studies with quantitative data allowed for statistical comparison of the data (e.g., (28, 34)), and the included qualitative studies were of low quality (e.g., (45, 46)). Furthermore, few studies compared computer-mediated interaction to face-to-face encounters, while no studies compared different technologies with each other.

Future research should describe the modalities used, situational factors, and end-user alterations in sufficient detail to determine the amount of filtering of verbal and non-verbal signals. In addition, studies that investigate how expressing emotions influence how an observer on the other end of the computer experiences these emotions and gives an emotionally supportive response are needed. Moreover, more in-depth analyses of how different verbal and non-verbal cues influence these steps of computer-mediated empathy are lacking.

## 5. Conclusions

Computer-mediated care is rising, especially in light of the current COVID-19 pandemic. Thus, research on how emotion is expressed and experienced and how to be emotionally supportive during online interactions is crucial. Currently, the study is too limited to draw general conclusions. Self-paced interventions seem to enhance emotion regulation, but research on emotional expression, experience, and support during online interactions is limited.

More in-depth analysis of computer-mediated interaction is needed, which involves looking at the effects of verbal and non-verbal cues and describing the modality used in detail, including any situational factors and end-user modifications.

## Data Availability

The original contributions presented in the study are included in the article/**Supplementary Material**, further inquiries can be directed to the corresponding author.
