# Supplementary material for: “Online, the counselor can't see me cry”: a systematic literature review on emotion and computer-mediated care
Source: Front Digit Health. 2023 Sep 1;5:1216268. doi: 10.3389/fdgth.2023.1216268 (PMC10504663; doi:10.3389/fdgth.2023.1216268)
Supplement: Supplementary file 1 [file Table1.pdf]

Supplementary Material

1 Supplementary Figures and Tables

1.1 Supplementary file 1: Table 1 Research Strategy

Research question: effect of computer-mediated-care on emotional expression, experience, emotional support and (long-term) emotion outcomes

Topic	Synonyms	Related term
eHealth	Online care, telehealth, telemedicine, telecare, telemental, telehomecare, remote care, mHealth	Online communication, videocalling, videoconferencing, instant messaging, internet forum, message board, chat room, e-mail, online communities, internet-based, web-based
Emotio* identification	Emotion recognition, affect recognition, affect identification, affect perception, emotion perception	Emotio* expression, affect expression, emotion regulation, affect regulation, emotional intelligence, mentaliz*, affect lexicon, emotion lexicon

1.2 Supplementary file 2: Table 2 The characteristics of the studies

**Authors (year)**	**Article title**	**Country**	**Study Design**	**Intervention(s) and comparator(s)**	**Sample size (N)**	**Participating population (psychosocial problems)**
Aho et al. (2012)	Mothers' experiences of peer support via an Internet discussion forum after the death of a child.	Finland	Longitudinal qualitative study design	1) Posting in private internet support group<br>2) No Comparator	4 mothers<br>631 messages	Bereaved mothers
Blank & Adams-Blodnieks (2007)	The who and the what of usage of two cancer online communities	USA / unclear (online message boards)	Mixed design	1) Posting on message board<br>2) No comparator	492 messages	Individuals posting on message boards about breast and prostate cancer
Boβ et al. (2017)	Efficacy of a web-based intervention with and without guidance for employees with risky drinking: results of a three-arm randomized controlled trial	Germany	RCT	1) Unguided web-based self-help<br>2) Web-based self-help with adherence guidance<br>3) Wait-list control	434<br>146 in unguided web-based self-help<br>144 in guided web-based self-help<br>144 wait list control	Males and females consuming at least 21 / 14 resp. standard units of alcohol a week and scoring $\geq$ 8/6 on the alcohol use disorders identification test
Brand et al. (2019)	An online educational program for individuals with dissociative disorders and their clinicians: 1-year and 2-year follow-up.	International (online)	Pre- post measurement by means of a survey	1) Online psychoeducational program<br>2) No comparator	111 patient – therapist dyads	Patient – therapist dyads with patients diagnosed with dissociative disorder
Chavooshi et al. (2016)	Telemedicine vs. in-person delivery of intensive short-term dynamic psychotherapy for patients with medically unexplained pain: A 12-month randomized, controlled trial	Iran	RCT	1) Internet delivered short term dynamic psychotherapy<br>2) In-person short term dynamic psychotherapy	81<br>39 internet-delivered<br>42 in-person	Patients with medically unexplained pain
Collie et al. (2007)	Videoconferencing for delivery of breast cancer support groups to women living in rural communities: a pilot study.	USA	Pilot / pre-post measurement (mixed methods)	1) Support group via videoconferencing<br>2) No comparator	27	Women with breast cancer
Czepczor-Bernat, et al. (2020)	The effect of a web-based psychoeducation on emotional functioning, eating behaviors, and body image among premenopausal women with excess body weight	Poland	RCT	1) Web-based psychoeducation with emotional functioning module based on Emotional Schema Therapy (EG I)<br>2) Web-based psychoeducation with emotional functioning module based on an eclectic approach (EG II)	129<br>43 in EG I<br>46 in EG II<br>40 in CG	Women with a BMI $\geq$ 25 kg/m²

				3) Wait list control group (CG)		
Doran, & Lewis (2011)	Posting incognito… Males with eating problems: Online emotional expression and support.	UK	Descriptive qualitative research	1) Posting in online platforms dedicated to males with eating problems<br>2) No comparator	15 males<br>101 messages in 19 threads	Individuals posting on an internet message board dedicated to males with eating problems
Döveling (2015)	Emotion regulation in bereavement: searching for and finding emotional support in social network sites.	Germany	Mixed methods (qualitative data which is quantified)	1) Posting in online bereavement platforms<br>2) No comparator	2127 messages from 318 users	Individuals posting in online bereavement platforms
Ebert et al. (2016)	Internet-and mobile-based stress management for employees with adherence-focused guidance: efficacy and mechanism of change.	Germany	RCT	1) Self-guided internet-based stress management intervention<br>2) Wait-list control group	264<br>132 in the intervention group<br>132 in the control group	Employees with elevated symptoms of perceived stress
Fonseca et al. (2019)	Be a Mom, a web-based intervention to prevent postpartum depression: The enhancement of self-regulatory skills and its association with postpartum depressive symptoms.	Portugal	RCT	1) Web-based intervention based on principles of cognitive behavioral therapy<br>2) Wait-list control	194<br>98 in the intervention group<br>96 in the control group	Women presenting with postpartum depression risk factors or early-onset postpartum depression
Foster (2016)	'Keep complaining til someone listens': Exchanges of tacit healthcare knowledge in online illness communities	Unclear (online message board, English speaking)	Combination of nonparticipant observation with archival analysis	1) Messages on online message board<br>2) No comparator	1655 messages (+observation)	Patients posting on online message board (among others Crohn's disease, rheumatoid arthritis and chronic pain)
Glück & Maercker (2011)	A randomized controlled pilot study of a brief web-based mindfulness training	Switzerland and Austria	RCT	1) Self-guided two-weeks online treatment group<br>2) Waitlist-control group	49<br>28 in treatment group<br>21 in waitlist control group	Adults
Köppe et al. (2019)	Improving Emotion Perception and Emotion Regulation Through a Web-Based Emotional Intelligence Training (WEIT) Program for Future Leaders	Germany	RCT	1) Online version of the EMO-TRAIN program<br>2) Wait list control	197<br>59 in treatment group<br>75 in waitlist control group	University students from business or business-related studies
Lewallen et al. (2014)	How language affects peer responsiveness in an online cancer support group: Implications for treatment design and facilitation	USA	RCT	1) Online support group<br>2) 12-week waitlist control group	525 discussion board messages posted by 116 participants in the health-space.net	Cancer survivors experiencing distress
Lieberman et al. (2003)	Electronic support groups for breast carcinoma: a clinical trial of effectiveness.	USA	Pre/post within-subjects design	1) 16 weekly electronic support groups in addition to a private newsgroup<br>2) No comparator	32 women	Women with breast carcinoma
Nicholas et al. (2004)	Communication features in an online group for fathers of children with spina	Canada	Qualitative study	1) Six-month online group<br>2) No comparator	25 fathers	Fathers of children with spina bifida

	bifida: Considerations for group development among men.					
Osma et al. (2016)	Multicomponent cognitive behavioral therapy for intermittent explosive disorder by videoconferencing: a case study	Zaragoza (Spain)	Case-study experiment	1) Cognitive-Behavioral Therapy via videoconferencing<br>2) No comparator	1	Male with intermittent explosive disorder, aged 33 years
Ruwaard et al. (2007)	E-Mailed standardized cognitive behavioral treatment of work-related stress: A randomized controlled trial	The Netherlands	RCT	1) 7-week standardized cognitive behavioral treatment of work-related stress conducted via e-mail<br>2) Wait list control	239<br>177 in treatment group<br>62 in waitlist control group	Participants who subscribed to this study on work-related stress
Setoyama et al. (2011a)	Benefits of peer support in online breast cancer communities: Differences between lurkers and posters	Tokyo, Japan	Exploratory, descriptive, cross-sectional, Web-based survey	1) Members of online breast cancer community<br>2) No comparator	465 people	Members of online breast cancer community
Setoyama et al. (2011b)	Comparing support to breast cancer patients from online communities and face-to-face support groups	Tokyo, Japan	Cross-sectional survey	1) Online communities<br>2) Face-to-face support groups	1039<br>127 only used online communities<br>374 used online communities and face-to-face support groups<br>538 only used face-to-face support groups	Patients with breast cancer
Tsaousides et al. (2017)	Improving emotion regulation following web-based group intervention for individuals with traumatic brain injury	USA	Pre–post study	1) Group treatment to improve emotion regulation via videoconferencing<br>2) No comparator	91	Individuals with traumatic brain injury and deficits in traumatic brain injury
Villani et al. (2018)	Promoting emotional well-being in older breast cancer patients: Results from an eHealth intervention	Online (unclear)	RCT	1) Two-week e-health stress inoculation training intervention<br>2) Control group: treatment as usual	29<br>15 in the treatment group<br>14 in the control group	Women with diagnosis of breast cancer with radical surgery
Weiss et al. (2018)	Telemental Health Delivery of Skills Training in Affective and Interpersonal Regulation (STAIR) for Rural Women Veterans Who Have Experienced Military Sexual Trauma	USA	Pre-post pilot Study	1) Ten weekly STAIR sessions via videoconferencing<br>2) No Comparator	Ten	Female veterans who had experienced military sexual trauma

Wright (2011)	Social support within an on-line cancer community: an assessment of emotional support, perceptions of advantages and disadvantages, and motives for using the community from a communication perspective	USA	Online survey	1) Use of online support groups<br>2) No Comparator	103	67 people indicated that they had cancer while 36 said they used the support groups because someone close to them had cancer.

1.3 Supplementary File 3 Data-extraction overview

1.3.1 Table 3a: Results related to expressing emotions

Author(s) Year	Description of the intervention	Medium of communication	Instruments used	Outcome on transmitting emotional cues	Other measurements
Aho et al. (2012)	Posting in private internet support group	Asynchronous text-based	Content analysis	Shared oppressive feelings included feelings of longing, guilt, bitterness, anger, fear, ambiguity, depression and bodily sensations.	Informational support and support through communality.
Blank, Adams-Blodnieks (2007)	Posting on message boards	Asynchronous text-based medium	Coding of messages	133 (19.3%) of the posted messages contained emotional expression. Somewhat more of these (72; 55.0%) were positive (e.g. expressions of well-being such as 'I'm getting through this') than negative (e.g. sense of losing control; 59%; 45.0%).	Who posted: self, spouse, immediate family, friend or other<br>Other categories of messages: medical/treatment, intimacy/sexuality, support, miscellaneous
Doran, & Lewis (2011)	Messages posted in online message board dedicated to males with eating problems	Asynchronous text-based	Coding of messages	1. Disturbed eating attitudes and linked emotions<br>• The majority of users expressed disturbed eating attitudes and behavior that were linked to negative emotions.<br>• Expressed emotions included disappointment, guilt, feeling fat, feeling ugly, misery, desperation, loneliness, depression, worthlessness, unhappiness, shame and suicidal ideation.<br>2. Being secretive<br>• New users tended to keep their eating problems a secret from their friends and family. This discouraged them to seek help.<br>• New users expressed using the board so they could express their feelings whilst remaining anonymous	Support (interrelated with emotional expression)
Döveling (2015)	Posting in online bereavement platforms	Asynchronous text-based	Coding messages on the basis of emotion regulation	Missed emotional support in the offline world accounts for 12.4% of all analyzed posts (situation selection). In adult platforms, the expression of missed support was less in two	Missed offline support

			patterns in social network sites:<br>• Situation selection: selecting online because of missed support offline<br>• Restoration-orientation: discussing other topics in long conversations	adult platforms (n=69, 9.3% & n=76, 12.3%) than in a youth platform.<br><br>Adult users predominantly address subject matters related to death, grieving and dying online, and touch only a small number of everyday topics in long conversations (9.7%) Predominant aspect in all forums was emotional communication (86%), with empathy (45.6%) and grief (31.7%) as the foremost emotions expressed online.	Feelings of social cohesion<br><br>Continuing online bonds with deceased
Foster (2016)	Posting on online message board	Asynchronous text-based	Analysis of content of messages	Members on the message board advise each other to refrain from becoming overly emotional. There is an acknowledged temptation to appear "desperate for relief" from symptoms as a means of communicating the debilitating effects of one's symptoms to one's clinician. Patients coach each other to engage in emotion regulation both during the meeting with the doctor, as well as leading up to the meeting. Many members worry that the ultimate consequence of being overly emotional is the outright termination of the physician-patient relationship by their doctors.	Information management<br>Treatment persistence
Lewallen et al. (2014)	The purpose of this study was to identify linguistic and qualitative characteristics of participants' messages that predict how other participants respond in an asynchronous discussion board for cancer-related distress	Asynchronous text-based	Emotional Expression and Personal Self-Expression using the LIWC2001	Original messages expressed more positive than negative emotions. Relative to the means of 43 studies analyzing LIWC samples, messages contained more positive emotional expression (3.9% vs. 2.4%) and an equal amount of negative emotional expression (1.7% vs. 1.6%). Messages high in positive emotions were less likely to receive a reply, OR=.94, p=.03. Use of negative emotions was not predictive of receiving a reply.	Exploration of topics
Nicholas et al. (2004)	Six-month online group. The group was facilitated by a social worker who provided minimal involvement in group dialogue beyond welcoming	Asynchronous text-based	Coding of messages	The use of narration and storytelling in men's groups appeared to be an effective tool in managing intense emotions and weaving in contextual details of individuals' lives.<br><br>Humor served to dissipate and temper strong emotive content. In some cases, fathers appeared to delay the emotional content	Use of a story genre<br><br>Use of information sharing for group formation

	group participants, stimulating discussion in 'quiet' times, and seeking to address technical problems associated with the online nature of the group			of what lay behind their experiences until they had been able to factually convey the precipitating and causal detail.<br><br>Conversely, too much unmodulated emotional content seemed to overwhelm and subvert group interaction and individual involvement.	Empowerment through information sharing<br><br>Fatherhood support; affirmation and cheering on
Setoyama et al. (2011b)	Online community for patients with breast cancer	Asynchronous text-based	Survey exploring participants' expectations of peer support resources	In the group using both face-to-face and online resources, support scores were compared. Emotional expression was higher for online communities (67.3) compared to face-to-face (51.3, p<.001).	Hospital Anxiety and Depression Scale [HADS] Disease-related characteristics<br><br>Use of online and face-to-face support groups

1.3.2 Table 3b: Results related to experiencing the emotions of the other user

Author(s) Year	Description of the intervention	Medium of communication	Instruments used	Outcome on feeling empathy	Other measurements
Lewallen et al. (2014)	The purpose of this study was to identify linguistic and qualitative characteristics of participants' messages that predict how other participants respond in an asynchronous discussion board for cancer-related distress	Asynchronous text-based	Emotional Expression and Personal Self-Expression using the LIWC2001	Messages high in positive emotions were less likely to receive a reply, OR=.94, p=.03. Use of negative emotions was not predictive of receiving a reply.	Exploration of topics
Nicholas et al. (2004)	Six-month online group. The group was facilitated by a social worker who provided minimal involvement in group	Asynchronous text-based	Coding of messages	Too much unmodulated emotional content seemed to overwhelm and subvert group interaction and individual involvement.	Use of a story genre<br>Use of information sharing for group formation<br>Empowerment through information sharing

	dialogue beyond welcoming group participants, stimulating discussion in 'quiet' times, and seeking to address technical problems associated with the online nature of the group				Fatherhood support; affirmation and cheering on

1.3.3 Table 3c: Results related to conveying emotional support

Author(s) Year	Description of the intervention	Medium of communication	Instruments used	Outcome on conveying empathy	Other measurements
Aho et al. (2012)	Posting in private internet support group	Asynchronous text-based	Content analysis	Emotional support included three categories:<br>1) Sharing oppressive feelings (see table 3 a).<br>2) Well-being was supported by showing compassion, comforting, wishing strength, sustaining hope, encouraging to take care of oneself and giving feedback.<br>3) Sympathy was displayed by showing involvement and being on someone's mind.	Informational support and support through communality.
Doran, & Lewis (2011)	Messages posted in online message board dedicated to males with eating problems	Asynchronous text-based	Coding of messages	1) Emotional support on the message board included expressions of encouragement, messages of good luck, compassion and understanding. Empathy was also expressed by validating users' feelings and expressions of other users feeling the same way, like a shared experience.<br>2) When users sought help after communication on the message board, they would share their experience and again get positive reactions. More regular users got to know each other online and provided regular encouragement	Support (interrelated with emotional expression)
Döveling (2015)	Posting in online bereavement platforms	Asynchronous text-based	Coding the content of messages	Predominant aspect in all forums was emotional communication (86%), with empathy (45.6%) and grief (31.7%) as the foremost emotions expressed online.	Missed offline support<br>Feelings of social cohesion<br>Continuing online bonds with deceased

1.3.4 Table 3d: Results related to being perceived as emotionally supportive

Author(s) Year	Description of the	Medium of	Instruments used	Outcome on being perceived as empathic	Other measurements

	**intervention**	**communication**			
Setoyama et al. (2011b)	Online community for patients with breast cancer	Asynchronous text-based	Survey exploring participants' expectations of peer support resources	Emotional support / Helper therapy was higher for face-to-face groups (73.5) compared to online groups (60.5, $p<.001$).	Hospital Anxiety and Depression Scale [HADS]<br>Disease-related characteristics<br>Use of online and face-to-face support groups
Wright (2011)	Online cancer support community	Asynchronous text-based	Emotional Support Scale	There was a modest negative correlation between perceived life stress (M = 18.6;SD= 6.62)and perceptions of emotional support (M= 65.10; SD = 6.80), $r(101)= -.19$, $p < .05$, $r^2 =.03$. In terms of the effects of experienced disadvantages of online communication on perceived emotional support; the ANOVA indicated significant differences between the groups, $F(7, 95) = 3.28$, $p < .01$. A Games-Howell multiple comparison procedure indicated that the individuals who reported, "not being able to hear tone of voice" as the most frustrating disadvantage of using the community had higher perceived emotional support scores (*M* = 69.68; *SD* = 2.72) than those individuals who indicated the most frustrating disadvantage was delayed feedback (*M* = 62.76;*SD* = 7.38),""flaming or off-topic (*M* = 63.15; *SD* = 7.00),"and "inability to detect deception (*M* = 59.88;*SD* = 7.27)."	Global Measure of Perceived Stress<br>Virtual support advantages and disadvantages.<br>Internet motives scale

1.3.5 Table 3e: Results related to (long-term) emotion outcomes

**Author(s) Year**	**Description of the intervention**	**Medium of communication**	**Instruments used**	**Outcome on results of being empathized**	**Other measurements**
Boβ et al. (2017)	The web-based intervention consisted of five modules and participants were advised to complete one module per week. Each module contained general information, illustrative examples, interactive exercises, quizzes, audio and video files, and downloadable work sheets.	Asynchronous communication mix	The emotional irritation subscale of the Irritation Scale	The combined guided and unguided web-based interventions reduced symptoms of emotional irritation compared to waitlist control both after 6 weeks ($d= .17$, $p<.001$) and 6 months ($d=.26$, $p<.001$)	Weekly standard units of alcohol consumption<br>Depression Anxiety Stress Scale<br>Irritation Scale<br>Effort Reward Imbalance Questionnaire – Short Form

	Emotional regulation techniques, that have not been tested in web-based alcohol interventions were integrated in the intervention.				
Brand et al. (2019)	The online intervention consisted of 45 short educational videos (5-15mins) and exercises (e.g. structured writing and behavioral practice exercises). The topics in the intervention were trauma related. Self-compassion and acceptance of emotions were key elements of the intervention.	Asynchronous communication mix (text and video)	Difficulties in Emotion Regulation Scale	Halfway the study:<br>• Overall small improvements in emotion regulation (*d*= 0.36)<br>• High dissociation group showed greatest improvement in emotion regulation (*d*=0.54)<br>• Low dissociation group showed small improvements in emotion regulation (*d*=0.26)<br>At study completion:<br>• Large improvements in emotion regulation (*d*=0.90)<br>• High dissociation group shows largest improvements in emotion regulation (*d*= 0.93)<br>• Low dissociation group shows medium to strong improvements (*d*= 0.32).<br><br>There was an interaction between emotion regulation difficulties and dissociative symptoms (F(2,107)= 3.95, p=.22). Patients with more dissociative symptoms showed more difficulties in emotion regulation at baseline, and a steeper decline in difficulties in emotion regulation compared to patients with less emotion regulation difficulties.	Dissociative Experiences Scale II<br>Posttraumatic Stress Checklist – Civilian Form<br>Progress in Treatment Questionnaire, patient version
Chavooshi et al. (2016)	16 individual hour-long sessions of graded intensive short-term dynamic psychotherapy (ISTDP), either in person or via videoconferencing. Key elements of ISTDP are emotional awareness and augmenting the capacities to self-reflect as well as tolerate and experience emotions.	Synchronous videoconferencin g	Emotion regulation questionnaire (ERQ)	In-person ISTDP showed higher cognitive reappraisal (95% confidence interval (5.1 to 13.7), p<.001) and lower suppression of emotion (MD=-3.8, 95% CI (-9.8 to 1.1); p<.001) compared to ISTDP via videoconferencing at 12-month follow-up.	Numerical Pain Rating Scale<br>Depression Anxiety Stress Scale-21<br>Quality of Life Inventory<br>Mindful Attention Awareness Scale<br>Treatment Evaluation Inventory Short Form<br>Psychotherapist Alliance Questionnaire
Collie et al. (2007)	A licensed clinical Social Worker led 8 2-h weekly support group sessions via	Synchronous videoconferencin g	Courtauld Emotional Control Scale (CECS)	No significant effect of the intervention on emotional expression from pre-test to post-test (*t*(16)=-0.44, *p*= ns, two-tailed	Stanford Psychosocial Oncology Questionnaire

	videoconferencing at a site nearby. Each woman was provided with informational material focused on key elements such as making good use of social support or emotional expression.				Centre for Epidemiologic Studies Depression Scale<br>PTSD Checklist-Specific Version<br>Cancer Behavior Inventory<br>Feasibility and experience with intervention via interview
Czepczor-Bernat, et al. (2020)	The web-based psychoeducation consisted of three modules: 1) emotional functioning module, 2) eating behaviors module, and 3) body image module. The content of the emotional functioning module differed between experimental groups. In one group this module was based on Emotional Schema Therapy. In the other group, the module was based on an eclectic approach based on compassion-focused therapy, acceptance and commitment therapy and dialectical behavioral therapy.	Asynchronous communication mix	Difficulties in Emotion Regulation Scale (DERS)	Both experimental groups increased in adaptive emotion regulation, $F(2, 109) = 9.67$, p<.001, $\eta_p^2 = 0.151$ They showed higher adaptive emotion regulation than wait list control on day 16 and on day 76, $F(4, 218) = 20.48$, p<.001, $\eta_p^2 = 0.273$.	Sociodemographic variables<br>Mindful Eating Scale<br>Positive and Negative Affect Schedule<br>Three-Factor Eating Questionnaire<br>Body Attitude Test
Doran, & Lewis (2011)	Messages posted in online message board dedicated to males with eating problems	Asynchronous text-based	Coding of messages	After many of the new users familiarized themselves with posting on the board, they felt less strongly about being secretive to friends and family and some considered seeking help, or actively sought it out.	Support (interrelated with emotional expression)
Ebert et al. (2016)	The intervention consisted of eight 45-60 mins modules, based on the distinction of problem-focused and emotion-focused coping. Participants could choose to receive automatic motivational text	Asynchronous communication mix (text and video)	Emotional exhaustion subscale of the Maslach Burnout Inventory (MBI - EE). Comprehension, acceptance and emotional self-	Emotion regulation skills and competencies (i.e., comprehension, acceptance, self-support and regulation of general distress) improved significantly more in the intervention group as compared with the control group, post treatment (resp *d*=.55, *d*=.59, *d*=.47, *d*=.78) and at 6 months follow up (resp *d*=.52, *d*=.68, *d*=.50, *d*=.68).<br>Repeated measures ANOVA revealed significant effect (F(1, 1.87)=30, p<.001) in favor of the intervention group for	Perceived Stress Scale-10<br>Centre for Epidemiological Studies' Depression Scale<br>Anxiety subscale of the Hospital Anxiety and Depression Scales<br>Insomnia Severity Index<br>Penn State Worry

	messages and exercises on their mobile phones. Participants were supported by an e-coach applying adherence-focused guidance.		support subscales of the Emotion Regulation Skills Questionnaire (ERSQ-27): Subscale regarding emotion regulation skills for general distress of the German Emotion Regulation Skills Questionnaire (using the Emotion Specific Version, ERSQ – ES – GD)	emotional exhaustion Follow-up simple effect showed significant effects for emotional exhaustion post treatment ($F(1,261)=48.17$, $p<.001$, $d=.76$) and at 6-month follow up ($F(1, 261)= 44.13$, $p<.001$, $d=.81$).	Questionnaire-Ultra Brief Version-past week<br>Utrecht Work Engagement Scale<br>Psychological detachment subscale of the Recovery Experience Questionnaire<br>Work loss days and work cut-back days with items from the German Version of the Trimbos and Institute of Medical Technology Assessment Cost Questionnaire for Psychiatry<br>German version of the Client Satisfaction Questionnaire
Fonseca et al. (2019)	Short-term, self-guided, web-based preventive intervention that targets women at risk for postpartum depression, or women with early onset postpartum depression. It integrates principles from cognitive behavioral therapy (CBT), including self-compassion and acceptance and commitment therapies. The program includes 5 modules. The content included psychoeducational information, practical strategies, interactive exercises with personal feedback and homework activities. Asynchronous communication channels were available to enable support with the program.	Asynchronous communication mix	The short version of the Difficulties in Emotion Regulation Scale (DERS-SF)	Levels of emotion regulation difficulties showed a greater decrease from pre to post-intervention in the intervention group ($\mu_\Delta = -4.35$, $p<.001$, $\sigma^2_\Delta = 87.96$, $p<.001$) than in the control group ($\mu_\Delta = -2.05$, $p<.001$, $\sigma^2_\Delta = 51.29$, $p<.001$) and this change was heterogeneous across individuals in both groups. The higher the emotion regulation difficulties before the intervention, the greater the decrease in emotion regulation difficulties after the intervention ($\sigma_{1\Delta} = -59.02$, $SE = 9.76$, $Z = -6.05$, $p<.001$).<br>There was a positive association between changes in emotion regulation and changes in depressive symptoms ($B=.115$, $SE=.038$, $p<.01$). A greater decrease in difficulties in emotion regulation levels was associated with a greater decrease in the levels of depressive symptoms.	Sociodemographic and clinical information<br>Portuguese version of the Postpartum Depression Predictors Inventory-Revised<br>Edinburgh Postpartum Depression Scale<br>Acceptance and Action Questionnaire-II<br>Short version of the Self-Compassion Scale
Glück &	The intervention consisted	Asynchronous	Emotion regulation	No effect of the intervention on emotion regulation.	German Brief Symptom

Maercker (2011)	of two six-day modules, containing 20mins mindfulness exercises. The training consisted of audio files, a flash animated exercise and written text. Techniques included awareness of body sensations, attention to breath and acceptance of upcoming emotions.	communication mix	(SEK-27)		Inventory<br>Symptom Checklist-90-R<br>German version of the Perceived Stress Questionnaire<br>German 14-item version of the Freiburg Mindfulness Inventory<br>Positive and Negative Affect Scale
Köppe et al. (2019)	The Web-based Emotion Intelligence Training (WEIT) program is based on the face-to-face EMO TRAIN training program. The WEIT consists of four one-hour modules and a four-week online practice period. It includes four consecutive modules, namely emotion perception in others, emotion perception in the self, emotion regulation in others and emotion regulation in the self. Various methods such as video clips, audio files or drag and drop exercises were used to create active learning experiences.	Asynchronous mixed media	Mayer-Salovey-Caruso Emotional Intelligence Test – Emotion Perception (MSCEIT – EP)<br>Mayer-Salovey-Caruso Emotional Intelligence Test – Emotion Regulation (MSCEIT – ER)	There was a significant group x time interaction for the MSCEIT EP, $F(1, 132) = 4.40$, $p = .04$. In comparison with the control group, the training group showed a significant decrease for the MSCEIT EP between times 1 ($M = 4.28$, $SD = 1.47$) and 2 ($M = 3.90$, $SD = 1.57$, $t = 1.96$, $p = .05$), representing an improvement of the ability to perceive emotions in oneself and others. No significant interaction was found for the MSCEIT ER $F(1, 132) = .08$, $p = .78$.<br>When looking at the long term effect, there was a significant group x time interaction for the MSCEIT ER, $F(1, 108) = 3.79$, $p = .05$, but not for the MSCEIT EP, $F(1, 108) = 1.67$, $p = .20$. The MSCEIT ER scores decreased significantly in both groups from time 2 (TG: $M = 5.03$, $SD = 1.49$, CG: $M = 5.16$, $SD = 1.33$) to time 3 (TG: $M = 4.16$, $SD = 1.56$, $t = 24$, $p = .00$, CG: $M = 4.72$, $SD = 1.34$, $t = 2.27$, $p = .03$), showing an improvement in the ability to regulate emotions in oneself and others in both cases. The training group showed larger improvements than the training group.<br>There was a significant decrease in MSCEIT EP for the control group from time 2 (M = 4.52, $SD = 1.74$) to time 3 ($M = 4.18$, $SD = 1.75$, $t = 2.63$, $p = .01$), revealing an improvement in EP. There was no significant difference in MSCEIT EP for the training group between time 2 (m = 3.96, $SD = 1.67$) and time 3 ($M = 3.87$, $SD = 1.77$, $t = .56$, $p = .58$), indicating that the improvement of EP remained stable after 6 weeks.	Irritation Scale

Lieberman et al. (2003)	Women participated in a closed electronic support group (ESG) for 16 sessions once a week. Sessions were mediated by a trained facilitator. The women also had access to a private online group in which they were free to post pictures, their cancer stories, chat and read transcripts of meetings they had missed.	Asynchronous, mainly text-based	The Courtauld Emotional Control Scale (CECS)	Participants showed higher emotional suppression after the intervention (t=4.2, p<.001).	The Centre for Epidemiologic Studies-Depression Scale<br>The Posttraumatic Growth Inventory<br>Pain: Self ratings, Intensity, Interference, and Reactions<br>Weinberger Adjustment Inventory<br>Mini-Mental Adjustment to Cancer Scale (short form)
Osma et al. (2016)	Mindfulness based Cognitive Behavioral Therapy via videoconferencing. The first three sessions were dedicated to face-to-face assessment. The remaining sessions were via videoconferencing. The first 8 sessions were administered weekly, the next 6 sessions every two weeks and the last 3 sessions were every three weeks.	Synchronous videoconferencin g	Emotional self-record (Linehan, 1993) providing information about the number of aggressive episodes, triggering stimuli, the patient's emotional response, and the consequences of his or her reactions.	Aggressive episodes declined progressively throughout treatment.	State-Trait Anxiety Inventory<br>Beck Depression Inventory<br>Revised NEO Personality Inventory<br>Anxiety Sensitivity Index<br>Positive and Negative Affect Schedule<br>The Rosenberg Self-Esteem Scale<br>Client Satisfaction Questionnaire
Ruwaard et al. (2007)	Self-paced 7 phases online cognitive behavioral treatment with therapist feedback via e-mail.	Asynchronous text-based	Emotional Exhaustion Subscale of the Maslach Burnout Inventory – General Survey	Both the treatment (d=.5) and control group (d=.2) showed significantly less emotional exhaustion at the post measurement. However, the treatment group improved significantly more (F(1, 236)= 8.8, p=.003, d=.3).	Depression Anxiety Stress Scales
Setoyama, et al. (2011a)	Lurkers and posters in online breast cancer communities	Asynchronous text-based	Self-developed survey measuring 8 categories of peer support, including emotional support and emotional expression.	For posters, emotional support/helper therapy (r = –.477, p < .001) was negatively correlated with the anxiety subscale. For lurkers, emotional expression (r = –.294, p < .001), was negatively correlated with the anxiety subscale. Emotional expression (r = –.116, p = .05) also had a slightly negative correlation with the depression subscale.	Hospital Anxiety and Depression Scale (HADS),<br>Participation in online communities<br>Disease-related characteristics<br>Received peer support
Tsaousides et al. (2017)	Group treatment to improve emotional regulation via	Synchronous videoconferencin	Difficulties in Emotion Regulation	Repeated measures MANOVA revealed significant improvement in DERS total score (F1.7,74 = 16.08, P < .001,	Positive Affect Negative Affect Schedule (PANAS

	videoconferencing to individuals with traumatic brain injury (TBI). Treatment was based on EmReg, a CBT-based intervention combining didactic and experiential training.	g	Scale (DERS),	η2 = 0.18, Greenhouse Geiser corrected). By the end of the 12-week follow-up, participants' average DERS Total scores decreased by 0.73 SD. DERS decreased from baseline to end-of-treatment (FT1-T2 = 14.18, P < .001, η2 = .16) and from the end-of-treatment to 12-week follow-up (FT2-T3 = 4.43, P=.04, η2 = 0.06), which indicates that participants continued to improve beyond the treatment period.	Satisfaction with Life Scale (SWLS)<br>Problem Solving Inventory (PSI)<br>Social Problem-Solving Inventory-Revised: Short Form (SPSI-R:S)<br>Dysexecutive Questionnaire (DEX)<br>Satisfaction with Therapy and Therapist Scale
Villani et al. (2018)	The e-health intervention comprised two phases skills acquisition and rehearsal (sessions 1–7) and application and follow-through (sessions 8–10). The skills acquisition and rehearsal aimed at providing patients with effective coping skills and techniques to manage potential negative emotions that might occur during chemotherapy. Participants viewed videos of woman having undergone chemo and practiced effective ways of coping with emotions.	Asynchronous communication mix	The Emotion Regulation Questionnaire (ERQ)	Emotional suppression scores decreased in the eHealth group from T1 (3.18) tot T2 (2.33), while they stayed the same in the control group (F(1,19)=5.5, p=.03). There were no significant effects for cognitive reappraisal.	The Functional Assessment of Chronic Illness Therapy – Breast
Weiss et al. (2018)	Treatment consisted of ten weekly sessions of STAIR delivered via videoconferencing, adapted for veterans. Key elements included psychoeducation on the impact of trauma on emotions and emotion regulation skills focused on increasing self-care and self-	Synchronous videoconferencin g	Difficulties in Emotion Regulation Scale (DERS)	Emotion regulation skills significantly improved DERS, F(1, 9) = 13.42, p = .005.	PTSD Checklist for DSM-5 (PCL-5)<br>Beck Depression Inventory-II (BDI-II)<br>Three subscales of the World Health Organization Disability Assessment Schedule 2.0 (WHODAS-2): Getting along with people, life activities and

	soothing activities.				participation in society<br>Client satisfaction questionnaire (CSQ-8)

1.4 Supplementary File 4 Quality assessment of the studies

1.4.1 Table 4 Checklist for assessing the quality of quantitative studies

**Criteria**	Boß et al. (2017)	Brand et al. (2019)	Chavooshi et al. (2016)	Collie et al. (2007)	Czepczor-Bernat, et al. (2020)	Ebert et al. (2016)	Fonseca et al. (2019)	Glück & Maercker (2011)	Köppe et al. (2019)	Lewallen et al. (2014)	Lieberman et al. (2003)	Osma et al. (2016)	Ruwaard, et al. (2007).	Setoyama et al. (2011a)	Setoyama et al. (2011b)	Tsaousides et al. (2017)	Villani et al. (2018)	Weiss et al. (2018)	Wright (2011)
Question / objective sufficiently described?	2	2	2	2	2	1	2	1	2	2	1	2	2	2	2	2	2	2	2
Study design evident and appropriate?	2	1	2	1	1	2	2	2	1	2	1	2	2	2	2	2	2	2	1
Method of subject/comparison group selection or source of information/input variables described and appropriate?	1	2	2	1	2	1	2	2	2	1	1	2	1	2	2	2	2	1	1
Subject (and comparison group, if applicable) characteristics sufficiently described?	2	2	1	2	2	2	2	2	1	2	1	2	2	2	2	2	2	2	2
If interventional and random allocation was possible, was it described?	2	N/A	1	N/A	1	2	2	1	1	N/A	N/A	N/A	2	N/A	N/A	N/A	2	N/A	N/A
If interventional and blinding of investigators was possible, was it reported?	0	N/A	2	N/A	0	N/A	N/A	N/A	0	N/A	N/A	N/A	N/A	N/A	N/A	N/A	2	N/A	N/A
If interventional and blinding of subjects was possible, was it reported?	0	N/A	N/A	N/A	0	N/A	N/A	N/A	0	N/A	N/A	N/A	N/A	N/A	N/A	N/A	N/A	N/A	N/A
Outcome and (if applicable) exposure measure(s) well defined and robust to measurement / misclassification bias? Means of assessment reported?	2	2	2	1	2	2	2	2	2	1	2	2	2	1	1	2	2	1	1
Sample size appropriate?	2	2	2	1	2	2	2	1	2	2	2	0	2	2	2	2	2	2	1
Analytic methods described/justified and appropriate?	1	2	2	2	1	2	2	2	1	2	2	1	2	2	2	2	2	2	1
Some estimate of variance is reported for the main results?	2	2	2	2	2	2	2	2	2	2	2	0	2	2	2	2	2	2	1
Controlled for confounding?	2	2	2	N/A	1	2	1	2	1	N/A	N/A	N/A	2	2	2	2	2	N/A	N/A
Results reported in sufficient detail?	2	2	1	2	1	2	2	2	2	2	2	2	2	2	2	2	2	2	1
Conclusions supported by the results?	2	2	2	2	1	2	2	2	1	2	1	2	2	2	2	2	2	2	2
**Total score**	**0,79**	**0,95**	**0,88**	**0,80**	**0,64**	**0,92**	**0,88**	**0,88**	**0,64**	**0,90**	**0,75**	**0,75**	**0,96**	**0,95**	**0,95**	**1,00**	**1,00**	**0,90**	**0,65**

1.4.2 Table 5 Checklist for assessing the quality of qualitative studies

**Criteria**	Aho et al. (2012)	Blank, & Adams-Blodnieks, (2007)	Doran, & Lewis (2011)	Döveling (2015)	Foster (2016)	Nicholas et al. (2004)
Question / objective sufficiently described?	2	2	1	2	1	1
Study design evident and appropriate?	2	2	2	1	1	1
Context for the study clear?	2	1	1	1	1	1
Connection to a theoretical framework / wider body of knowledge?	1	2	1	2	2	1
Sampling strategy described, relevant and justified?	1	2	1	0	1	1
Data collection methods clearly described and systematic?	2	2	1	0	1	1
Data analysis clearly described and systematic?	2	1	1	0	2	0
Use of verification procedure(s) to establish credibility?	0	0	2	0	0	0
Conclusions supported by the results?	2	1	2	1	1	1
Reflexivity of the account?	2	0	1	0	0	0
**Total score**	**0,80**	**0,65**	**0,65**	**0,35**	**0,50**	**0,35**
